# Can portable air quality monitors protect children from air pollution on the school run? An exploratory study

**DOI:** 10.1007/s10661-020-8153-1

**Published:** 2020-02-21

**Authors:** James Heydon, Rohit Chakraborty

**Affiliations:** 1 University of Nottingham, Nottingham, UK; 2 University of Sheffield, Sheffield, UK

**Keywords:** Air pollution, Air quality, Portable monitors, Coping theory, Environmental risk

## Abstract

With air quality issues in urban areas garnering increasing media attention, concerned citizens are beginning to engage with air monitoring technology as a means of identifying and responding to the environmental risks posed. However, while much has been written about the accuracy of this sensing equipment, little research has been conducted into the effect it has on users. As such, this research deploys coping theory to explore the specific ways in which portable air quality sensors influence user behaviour. This is done using a qualitative exploratory design, targeting parents and carers of children on the school run. Drawing from survey and interview responses, the article illustrates the decision-making pathways underpinning engagement with monitors and the ways in which they influence beliefs and behaviours around air pollution. The study demonstrates that personal environmental monitors can play a role in protecting children from air pollution on the school run. They can raise awareness about air pollution and disrupt misconceptions about where it does and does not occur. They can also encourage the public to change their behaviour in an attempt to mitigate and manage risks. However, the findings additionally reveal that sensor technology does not generate a simple binary response among users, of behavioural change or not. When attempts at behavioural change fail to reduce risk, resulting negative feelings can lead to inaction. Hence, the relationship between the technology and the individual is entwined with various social circumstances often beyond a parent or carer’s control. Thus, top-down support aimed at tackling air pollution at source is essential if this bottom-up technology is to fulfil its full potential.

## Introduction and background

Urban air pollution is one of the most pressing concerns for governments worldwide, with the United Nations calling on national and subnational governments to commit to achieving air quality safe for citizens by 2030 (United Nations 2019). Linked to around 40,000 premature deaths each year in the UK alone (Royal College of Paediatrics and Child Health 2016: xiii), air pollution poses several other risks to human health. Both long- and short-term, high- and low-level exposures are associated with adverse effects (Clifford et al. 2016), particularly when considering that for one of the main components in polluted air—particulate matter—there exist no “safe” levels (Qian et al. 2017). Ambient, or outdoor, air pollution is associated with increased rates of lung cancer, emphysema, bronchitis and other respiratory infections, with traffic-related air pollutants being suspected of initiating “diverse lethal diseases to considerably reduce life expectancy” (Kim et al. 2017: p. 277; Kelly and Fussell 2015). A “significant association” has been found between particulate matter 2.5 (PM 2.5) exposure and stroke, dementia, Alzheimer’s disease, autism spectrum disorder and Parkinson’s disease (Fu et al. 2019: p. 1240). As highlighted by a global review into the human health consequences of air pollution, lungs and airways notwithstanding, air pollution damages “most other organ systems in the body” (Schraufnagel et al. 2019: p. 417).

The risks to children are particularly pronounced because they tend to be exposed to higher concentrations than adults. Not only are their immature and developing immune systems and lungs implicated, along with their relatively high inhalation rate (Gehring et al. 2013; Kim 2004), but children also spend more time outside, and often walk or are pushed in buggies, usually at the height of exhaust emissions (Kenagy et al. 2016). Cognitive development among primary school children is also negatively affected, particularly working memory and attentiveness (Forns et al. 2017), with air pollution also being linked to the onset of neurological disorders. This includes autism and attention deficit/hyperactivity disorder (Myhre et al. 2018), and is associated with decreases in the protein important to cognition and the white matter involved in learning and brain function (Sram et al. 2017; see also Bandyopadhyay 2016). Such effects continue across the life course; “[t]he evidence to date is coherent in that exposure to a range of largely traffic-related pollutants has been associated with quantifiable impairment of brain development in the young and cognitive decline in the elderly” (Clifford et al. 2016: p. 383).

In the UK, these risks have long been recognised by the government. However, since 2011, its efforts at reducing air pollution have been repeatedly deemed inadequate by the courts. In the latest decision, the High Court described government countermeasures as “unlawful” ([2018] EWHC 315 (Admin) Case No: CO/4922/2017, para 118), while a cross-party inquiry concluded that “[t]he Government cannot continue to put public health at risk” by continuing to pursue ineffective countermeasures (The Environment, Food and Rural Affairs Committee 2018). The degree of inaction taking place gains prominence when recognising that outdoor air pollution exceeds World Health Organisation (2016) limits for 90% of the UK’s population. It is a situation that caused the UN Special Rapporteur on the human rights implications of hazardous substances, Baskut Tuncak (UN Human Rights Council 2017: 9), to express “alarm that despite repeated judicial instruction, and recommendations by the UN Committee on the Rights of the Child, the government “continues to flout its duty to ensure adequate air quality and protect the rights to life and health of its citizens”.

As a consequence of this dormancy, citizens have to rely on small networks of official monitors that are often unable to capture the complexity of urban air pollution (Paulos et al. 2009). Major UK cities have few monitors with publicly available data relative to their populations. For instance, despite Manchester, Leeds and Liverpool having populations of around half a million, each only contains two such monitors. With a similar population size, Sheffield only has three. As such, individuals and community groups are increasingly making use of an emerging technology—relatively low-cost personal air quality monitors—to evaluate environmental risks and respond accordingly (Marsh 2017). As Oltra et al. (2017: p. 296) note, the number of citizen-led, participatory sensing projects has “increased significantly in recent years”, with several companies bringing this emerging technology to market. These include the Plume Labs “Flow”, CleanSpace Tag and Atmotube Pro, to name a few. While much has been written about the accuracy of the equipment, in terms of its ability to contribute to official monitoring programmes, a recent systematic review of the literature concluded that “[c]urrently, there are very few studies that evaluate [its] social or economic implications” (Hubbell et al. 2018: p. 887). It is directly in this gap that the present research is situated.

The few studies that do exist are insightful, providing preliminary conclusions to suggest that personal exposure information can challenge preconceptions and inform people’s responses to air pollution. In relation to perceptions and emotions, the reported effects of monitor use range from an increased awareness of air pollution and intense emotional reaction (Oltra et al. 2017), through to enjoyment and surprise (Bales et al. 2019; Wong-Parodi et al. 2018; Bales et al. 2012). In relation to behaviour, Wong-Parodi et al. (2018) and Zappi et al. (2012) found minor behaviour changes to occur, which include actions like closing windows and ceasing to burn incense indoors, while Oltra et al. (2017) did not witness any alteration—intentional or real—as a result of monitor use. An overview of these studies can be seen in Table 1.

**Table 1:** Existing studies on behavioural responses to personal air quality data

Study	Focus	Monitor use duration	Sample size	Sample constitution
Zappi et al. (2012)	Human responses to air quality data on the commute	Between 2 and 4 weeks	16	On-campus workers
Bales et al. (2012)	Human responses to air quality data on the commute	4 weeks	16	On-campus workers
Oltra et al. (2017)	Comparing human responses to personal air quality data with traditional information sources	7 days	12	Selected according to demographic and socio-economic characteristics
Wong-Parodi et al. (2018)	Human responses to indoor air pollution responses	3 weeks	4*	Selected according to library users that borrowed an indoor pollution monitor
Bales et al. (2019)	Human responses to air quality data on the commute	4 weeks	29	Group 1: On-campus workers Group 2: On-campus workers from the same building
Heydon and Chakraborty (2020)	Adult responses to air quality data on the school run	2 weeks	45	Self-selection of parents and carers

^*^26 participants were surveyed after using an indoor monitor, but the findings indicate that the specific data on perspective and behaviour change was derived from the 4 of these that agreed to be subsequently interviewed

While insightful, these studies collectively exhibit several limitations. First, little attention is given to the specific mechanics of the transformative process. It is therefore not understood how the data intersects with individual beliefs and thought processes on the way to generating a given response. Second, they are overwhelmingly atheoretical, meaning that analyses are not informed by a sufficiently detailed understanding of how human beings react to stressful encounters, such as that elicited by exposure to high levels of air pollution. Third, none of the existing studies is sensitive to context, neglecting to account for any external factors which may influence the possibilities for change. This leads to a one-dimensional account of how monitors may affect behaviour. Finally, there are a myriad of shared shortcomings pertaining to the methodologies deployed; the majority of existing studies draw on relatively small and homogenous samples, while Zappi et al. (2012) and Bales et al. (2019; 2012) use figures from the same study conducted almost a decade ago, although the latest iteration is augmented with more recent data.

In light of these limitations, the purpose of this article is three-fold. First, it explores the extent to which perceptions and behaviours around air pollution on the school run are altered by personal exposure information. Second, it deploys Lazarus and Folkman’s (1984) coping theory to draw out the specific decision-making processes by which this transformation does or does not occur. Almost 40 years from its publication, this theory “remains the cornerstone of psychological stress and coping research across multiple fields and disciplines” (Biggs et al. 2017: p. 361). Third, by providing parents and carers on the school run with personal monitoring technology, its use is situated in the real world. This allows for the intersections between structure and agency to be observed and enables individual coping processes to be understood in context. Taken together, this study aims to provide further information on the transformative potential of emerging technology in a context of heightened environmental risk. In doing so, it seeks to illustrate some of the opportunities and barriers that relate to personal environment monitors and draw out the extent to which they can be used to protect some of the most vulnerable populations in society from the risks of air pollution.

## Theoretical lens

Given the lack of theory guiding existent research on the social implications of personal air quality monitor use, direction can be taken from research into the adjacent area of wearable healthcare devices. Much of this literature is concerned with determining the “infusion” of this technology into a person’s life and the various barriers to that (see Casselman et al. 2017; Evenson et al. 2015). For instance, concerns around health and privacy risks have been found to inhibit use (Piwek et al. 2016; Mills et al. 2016). However, similar to the research on personal environment monitors, little detailed information exists on the specifics of this encounter. To address this limitation, Marakhimov and Joo (2017) applied coping theory to wearable healthcare devices to ascertain the specific ways in which people respond to the concerns presented by the technology. In much the same manner, but using an explorative qualitative orientation, this study uses coping theory to understand how adults respond to personal air quality data on the school run.

Originally developed by Lazarus and Folkman (1984), the process-based model of coping explains how a person evaluates and responds to stressful encounters. It is “process-based” because coping is not conceived as a personality trait, referring instead to an unfolding and iterative relationship between person and environment. As such, “coping” refers to the “thoughts and behaviours used to manage the internal and external demands of situations that are appraised as stressful” (Folkman and Moskowitz 2004: p. 745). Internal demands are conceived of as personal factors, encompassing commitments and beliefs, while external demands pertain to the more contextual properties of events themselves. Importantly, the “extent to which any event is stressful is determined by a confluence of person and situation factors in a specific transaction” (Lazarus and Folkman 1984: p. 83).

The theory posits that a chosen coping behaviour is based on an initial two-part appraisal of a given encounter with environmental stimuli. During a “primary appraisal”, a person evaluates the impact of the event on his/her personal well-being, with the transaction being deemed positive, irrelevant or stressful (Biggs et al. 2017). It is here where the meaning attached to what is at stake holds importance as it influences how stress appraisals are categorised. Explaining this further, Lazarus and Folkman (1984: pp. 32–33) note that such stress may be regarded as a form of “harm/loss”, where some damage to the person has already been sustained, as a “threat”, which involves harms or losses that have not yet taken place but are anticipated, or a “challenge”, which is relatively positive and focuses on the potential for gain or growth. Also implicated at this stage is the “secondary appraisal”, where a person evaluates the extent of their control over the stressor to determine what can be done to manage it or mitigate its consequences. These two forms of appraisal are not sequential, but work upon one another to produce a perception of the situation:

*When that which is at stake is meaningful and coping resources are judged less than adequate for managing the demands of the situation, psychological stress is experienced. The greater the imbalance, the greater the stress.* (Folkman 1982: p. 97)

Taken together, this initial bifold stage of “cognitive appraisal” is important because “an individual’s appraisal of the situation greatly influences their resultant emotions, coping strategies, and subsequent outcomes” (Biggs et al. 2017: p. 353). Indeed, it is only following this stage, where an individual encounters a given stimuli, conceptualises it as stressful or not and decides what can be done to manage it, that actual mental and behavioural adaptations are engaged.

A myriad of coping responses can result from this antecedent stage, but they can be grouped into two distinct but related categories: problem-focused and emotion-focused coping (Lazarus 2006; Lazarus and Folkman 1984). Problem-focused outcomes refer to individual attempts at managing or mitigating the source of the stress. Emphasising proactive attempts at altering the situation, this includes efforts at reducing or removing obstacles, attaining new skills, planning, taking action and seeking assistance (Lazarus and Folkman 1984). By contrast, emotion-focused responses regulate emotions, referring to internal attempts at mitigating the emotional distress brought on by the stressful event (ibid). Writing just before his death, Lazarus (2006: p. 22) bemoaned the rigidity of these categories, noting that “it would be desirable to abandon the idea” of their independence from one another. Instead, research should acknowledge that in reality, they “operate together as a coherent unit and to separate them and set them up as competitive is to distort the way coping actually works” (ibid: p. 23).

Following this process of cognitive appraisal and coping effort, individuals may initiate a “reappraisal” in order to ascertain whether a given problem- or emotion-focused coping response was effective at mitigating the stress experienced. It refers to a new process of appraisal following an earlier one but, in essence, “appraisal and reappraisal do not differ” (Lazarus and Folkman 1984: p. 38). “Reappraisal” therefore converts what appears to be a linear process into a circular one, acknowledging that an initial coping response can alter a subsequent appraisal of the situation.

To conceive of individual responses to environmental stimuli in this way, as a process through which meaning is attributed to a given situation and then acted upon or not, draws attention to the specific cognitive and behavioural stages involved and the relationships between them. In doing so, coping theory provides a more nuanced and informed framework for analysis when compared with the atheoretical approach adopted by all existing scholarship on personal environmental monitor use. Taking this as the point of departure, the article now turns to the application of this framework to personal air quality monitors, with the aim of understanding how adults on the school run respond to the data encountered.

## Methods

### Research design

The design of this study is exploratory and qualitative. The data was collected from surveys and interviews, and the sample of parents and carers was drawn from 15 primary schools across Sheffield, England. With the primary aim of exploring the extent to which perceptions and behaviours of air pollution are altered by personal exposure information, the surveys were administered prior to receipt of the portable sensors. Participants were then asked to use the monitors for 2 weeks on the school run before being interviewed about their experiences, perceptions and behaviours during this time. The fieldwork was conducted between April and July 2019.

### Sample

The sample consisted of 45 participants, the average age of which was 42. Thirty-eight of these were female and 7 male, reflecting the gendered nature of the school run journey more broadly (Jain et al. 2011). Participation was limited to parents and carers that do the school run journey to a primary or infant school. Owed to the consumerist nature of the monitoring technology in question, participation was based on self-selection. As understanding real-world engagement with the technology was of priority, and those most concerned about air pollution are also most likely to purchase such monitors, this approach was deemed consistent with the purpose of the study. Indeed, 84% (*n* = 38) of participants responded with “strongly agree” or “agree” when surveyed about the extent of their agreement with the statement “air quality is a problem on my school run”. Participants were recruited through school newsletters and social media.

### Procedure

Following the initial expression of interest, participants were sent an electronic copy of the survey to complete. Qualtrics was used for this purpose. Arrangements were then made to hand over the monitor. Upon meeting, one of the research team would link the participant’s mobile phone to the specific monitor, explain and demonstrate how it and the accompanying app work, and address any questions. They were then instructed to use the monitor on the school run for 2 weeks and to check the monitor and app during this time. Interviews were conducted at the conclusion of this period, with questions centring on participant psychological and behavioural experiences over this time. To facilitate reflection on aspects of change within this experience, specific survey responses given by each participant were also recalled during the interview: those reflecting the degree with which participants considered air pollution to be a problem on the school run, their level of concern about the issue, knowledge of pollutants, and their origins and health effects. Interviews were conducted by an experienced social researcher.

The measurements were taken with a Plume Labs “Flow” air quality monitor, which senses PM_2.5_, PM_10_, NOx and VOCs. The unit weighs 70 g and the charge lasts approximately 24 h. The monitor displays four colours depending on the quality of air being measured. Using guidelines established by the World Health Organisation and U.S. Environment Protection Agency, these include green for “low” air pollution, yellow for “moderate”, red for “high” and purple for “very high”. The app itself provides more details, displaying the real-time air quality index figures to which the colours correspond. This particular unit is also linked to a mobile phone, using its Global Position System capability to plot these colours on a map according to where the user travelled. No restrictions on use were introduced, only a minimum requirement to use the monitor on the school run over 2 weeks. No substantive changes were made to the monitoring unit or graphical user interface over this time.

### Analysis

The interview transcripts were thematically analysed according to the framework established by Braun and Clarke (2006). This was conducted alongside the data collection stage, allowing participant recruitment to continue until no new or deviating data was being added to the categories of analysis. This is a standard akin to theoretical saturation, but without the framework of grounded theory (see Saunders et al. 2017). The analysis allowed for both inductive and deductive codes to be generated, although those pertaining to the key stages of coping theory formed primary focus. The qualitative analysis was conducted using NVivo, while the quantitative survey data was exported from Qualtrics and analysed using SPSS.

### Limitations

The study draws its data from participants with specific and substantive constraints on their time. As parents and carers, most were managing several substantive responsibilities, meaning that findings may differ for those in situations with fewer constraints. The majority of participants were also women, meaning that there may be a gendered dimension to monitor engagement. Finally, a feature of the primary and infant school system in the UK, 44 of the 45 participants lived within 3 miles of the schools. As such, results may differ among those with a longer school run journey time. Ultimately, further study is required into whether the findings are replicable across different groups.

## Findings and analysis

The data gathered illustrates that a large proportion of participants engaged in problem-solving efforts, attempting to change their behaviour as a means of mitigating the perceived “threat” of air pollution. These were then reappraised to see if said changes were effective. A second, smaller group of participants followed a different process, pursuing efforts affiliated with emotion-focused coping without first attempting behavioural alteration. Changes in general and specific beliefs about air pollution were also reported, which were common across the sample and did not depend on the specific coping efforts deployed. Each of these aspects is taken in turn, following the structure of coping theory.

### Primary and secondary appraisal: threat and agency

Primary appraisal ascribes meaning to a specific transaction, determining the significance of that to an individual’s well-being (Lazarus and Folkman 1984). The transaction may be deemed positive, irrelevant or stressful. For the majority of participants (*n* = 40), the monitors heightened or confirmed pre-existing perceptions of air pollution as a problem on the school run. For 40% (*n* = 18), air pollution was seen to be more of a problem than originally thought, prior to use of the monitor, whereas most of the others reported having their preconceptions confirmed. Four people no longer thought that air pollution was a problem on their school run and 1 remained uncertain. Following this recognition, the majority (*n* = 41) of participants defined their encounter with air pollution readings negatively and in terms of anticipated harm. This conforms to Lazarus and Folkman’s (1984) concept of “threat”, which refers to an expected loss. There was parity of perception across the group; those reporting mainly “purple” and “red” readings exhibited the “threat” response, while those that did not encountered mainly “amber” and “green” readings. All those whose primary appraisal was defined by “threat” anticipated loss in terms of the health of children, and not only their own:

*When the kids were involved it’s definitely a lot more emotional. And I’m more angry, because I’m like ‘for fucks sake, this is no good. I don’t want this going into my kids’ lungs’. You see people pushing babies around and stuff like that and, you know, it’s like ‘oh god’. When our kids were in buggies and prams and they are right down there, they are right at the front, at the crossing lights and things like that. And you do get a bit of guilt, thinking ‘crikey, what have I done?’.* (Participant 9)

*I worry about the long-term effects on me and my kids, my wife and everyone…and the fact that the schools are there, so all the kids...And there’s no barriers – at the junior’s or the infant’s – between them and the road, so it’ just goes straight in.* (Participant 20)

*I’m worried. I’ve got school kids, obviously, but I’ve also got 20 month and 9-month-old children and I’m pushing them along at exhaust level…and while the pavement is quite wide often there’s traffic blocking the roads and it’s queuing at their level.* (Participant 41)

As can be seen, throughout the interviews, air pollution was primarily interpreted as a threat to the health of children, an unsurprising characteristic given that participants were selected on the basis of their school run journey. Yet, this is relevant because the meaning attached to a given encounter has an influence on the secondary appraisal, what individuals think they can do to manage the stressor and its associated distress (Dewe and Cooper 2007). Of the 41 that defined air pollution as a “threat”, 63% (*n* = 26) believed that they had the capacity to make behavioural adjustments sufficient to mitigate it. For these, this balance between primary and secondary appraisal led to various attempts at problem-focused coping.

### Problem-focused coping

When a situation is deemed to be stressful, requiring efforts to manage or resolve it, coping actions are enacted (Lazarus and Folkman 1984). In this regard, 26 participants altered their behaviour as a result of the monitor data. The main change attempted was to try alternate routes to and from school, away from the main roads; 16 attempted this. Six participants also used their car less, while 4 reported asking people to turn off their engines if seen idling outside school:

*I think that’s one thing that’s come out of using the sensor. Almost every day I’ve had to ask someone to turn off their engine. The thing that’s really…I’ve never been the sort of person to ask a stranger to do something because I feel like it’s a bit presumptuous and I do not like being…I just do not like telling people what to do, I’m not a very confrontational person. But I feel like if I do not, I do not know how long they are going to sit there idling their car and I’m looking at the monitor and thinking ‘it’s getting red, it’s red!’, and we are breathing in all the particles…so since having the monitor it’s made me that little bit…it’s given me the courage to ask people to turn their engines off.* (Participant 7)

*I am trying to use the car less. I probably would have driven probably four times a week to school…but we are trying not to drive at all now. We maybe drive once a week. It has changed quite drastically for us, so that we leave the car at home.* (Participant 14)

*Me and one of my friends have consciously not walked along [the main road] as much to get to school and back, so we’ll go right round the back streets a bit more.* (Participant 34)

Upon enacting these changes, participants believed that improvements in the quality of air would be visible, thereby easing their stress. It is here where, for the 26 who attempted problem-solving strategies, early engagement with the monitor reflects a very specific secondary appraisal, one reliant on the assumption that individuals can alter their situation to avoid air pollution and minimise the “threat” envisaged.

Air pollution in urban areas is, however, complicated, entailing dynamic and complex interactions between natural and anthropogenic environmental parameters. Twenty of the 26 that attempted problem-focused coping soon experienced this complexity, with the monitoring data recording minimal changes to their exposure despite the behavioural changes adopted:

*It just felt like every route was bad or good depending on the day. I did not feel like there was a solution. And we literally only have two routes, so there’s not really many options.* (Participant 6)

*Me changing my route at this specific time might work this week, but next week I might get different readings. It might be a bit of a, not a waste of your time, but a waste of your mental capacity trying to actively dodge things…One thing that these apps show you is that it might not matter where you are.* (Participant 12)

*I hoped it would change because the routes are pretty bad, and if there were clear differences – if one was green, one was yellow and one was red – then I would definitely take one over the other, but they have been alternating. So you get ones higher on some days and others higher on other days. It’s hard to know, really.* (Participant 22)

The 15 participants who perceived air pollution as a “threat” but did not act moved straight onto coping efforts associated with emotion-focused coping (see below), but those who attempted behavioural changes moved into the stage of “cognitive reappraisal”. Here, the effect of any changes made was re-evaluated to see if they had addressed the initial “threat” perception.

### Cognitive reappraisal: powerlessness and heightened threat

Participants who had changed their behaviour entered the reappraisal stage of coping expecting to see a difference in the levels of air pollution encountered. However, the expected improvements tended not to be reflected in the monitoring data. As such, when participants reappraised the situation following their behaviour changes, the over-riding feeling was very different to that felt during the initial appraisal. They now started to feel powerless:

*I felt really, really, really rubbish. Verging on depressed. Questioning my life choices. Really sad and powerless because we are not in a position where we can move house or just buy an electric car. We’re just not in that financial position. So, powerless, upset and ignorant as well. ‘How come I didn’t know this before?’. ‘Why is it not on the weather forecast every day?’.* (Participant 4)

*Something that’s become quite apparent is that you cannot actually avoid the pollution. And that’s it. You cannot free my or the children that live in my community from the air pollution. They live too close to dirty roads. It’s a bit depressing really.* (Participant 17)

*I was just thinking ‘I want to move’ actually. I was seeing the readings outside the house and you think ‘well, I still have to take the kids to school’. You feel like there’s no escape from it.* (Participant 26)

*I suppose it’s a powerlessness. I know that [the road to school] is awful and that’s where I walk my children to twice a day and that’s where they go to school, and on a hot day they have their windows open and it’s all just going in. From my point of view it’s been pretty bad because it’s confirmed that ‘oh no, it’s really bad and I knew it was really bad, and now I know it’s definitely bad’. So yeah, a powerlessness on the day to day level.* (Participant 29)

At the point of reappraisal, the same formula holds as for an initial appraisal; the difference between “primary” and “secondary” appraisal influences the degree of “threat” felt. As such, at this point, the monitors had not only served to reveal how poor the air quality on the school run was, thereby increasing awareness about the already-present concern for their children’s health, but also demonstrated that their ability to avoid it—either through behavioural changes or seeking refuge in perceived “sanctuary spaces” (see below)—was constrained by factors beyond their individual agency. As a result, the definition of their encounter with air pollution data did not change from the “threat” designation, but heightened it:

*I would not say it’s kept me up at night, but there were times when I’d wake up at night and I’d be thinking about it…it’s kind of taking over. I’m really thinking about this all the time.* (Participant 1)

*I think it’s even worse than I thought it was, which is really scary. Really scary.* (Participant 18)

*I think I’m more concerned now, if I’m honest. I would say very concerned. It’s pretty bad at times.* (Participant 22)

This response was common across the sample of participants. After using the monitors, 44 of the 45 participants reported concern with the levels of air pollution on the school run, with just over 70% (*n* = 32) being either “extremely concerned” or “very concerned”. Forty percent (*n* = 18) of participants thought that it was more concerning than originally thought, while 18% (*n* = 8) experienced a decrease but still retained some degree of concern. The 4 participants who thought air pollution was “no longer a problem” are not all mirrored in the figures for the “unconcerned” category because 3 noted that, while they now saw their school run as pollution-free, they were still concerned about other children on other routes.

### Emotion-focused coping: resignation

As the feelings of powerlessness became increasingly embedded with each cycle of attempted behavioural change, the appraisal-reappraisal feedback loop tended to result in feelings of resignation. This did not supplant problem-focused efforts, where they were attempted, but were instead experienced alongside them. This accords with Lazarus’ (2006) call for the two to be viewed not as mutually exclusive, but entwined. Other expressions thematically related to this category were reported, including sadness, helplessness and acceptance, but the over-riding form of emotion-focused coping communicated at the conclusion of the study was this notion of resignation:

*I was comparing it to somebody to Brexit, where you might have been really passionate at the start of Brexit, but by now you are just like ‘I don’t care anymore’. With this, it’s kind of like ‘well, I really want to make some good choices but, actually, everything is bad; there’s nothing I can do’. So, that’s been a bit of a shame* (Participant 3)

*I think initially I was more frustrated and scared but as it goes on you get used to what’s going on and you are not surprised by what’s happening, so I guess the feelings dissipate a little bit. That’s probably what happened. ‘Oh, there it is again. Yeah, that’s what I expected’.* (Participant 11)

*You get this data, you go ‘okay’, then it just makes you feel a bit worse.* (Participant 12)

*I became resigned. I am only one ant in the grand scheme of things and, really, what can I do to change anything?* (Participant 18)

*I felt sad and a bit helpless because at the beginning we were getting some green readings and I thought it would maybe never turn green on this road. And there was a couple of school runs that we did, earlier, that were green, and I felt a little glimmer of hope like ‘oh, this isn’t as bad as I thought it was’. But then, actually quite quickly it turned to where I cannot actually remember the last time I saw a green reading for it.* (Participant 21)

Such expressions of resignation were not limited to those pursuing behavioural adjustments, but were also reported by many of those that did not make changes. Here, a dovetailing of the various coping efforts can be witnessed, where the use of personal air quality monitors eventually resulted in resignation irrespective of whether behavioural change had been made at some point or not. This occurred for two reasons. First, as noted above, avoiding air pollution in densely populated urban areas, particularly at peak times of travel, is complicated. Distance-decay gradients differ depending on wind direction (McConnell et al. 2005; Gilbert et al. 2003); upwind, particulate concentrations can fall to near background levels within 200 m, but downwind concentrations do not reach background levels until 300–500 m. In some studies, this is extended to 800 m for ultrafine particles (Reponen et al. 2003) and 1500 m for NO_2_ (Gilbert et al. 2003; Jerrett et al. 2007). This is further complicated by the basis of these figures on patterns of motorway pollution; they do not account for settings where other sources of emissions are in close proximity, which is the reality of urban areas.

Second, the context in which the technology is used has a bearing on the options available for behaviour change. Seventy-five percent (*n* = 34) of participants described their feelings of resignation in relation to real-world circumstances serving to inhibit their ability to alter behaviour in ways they thought would be of benefit. The two primary constraints mentioned pertained to time, as manifest through responsibilities to school, work or family, and space, including a lack of route alternatives or availability of safe, efficient and affordable transport options. For those who did not make behavioural changes, these circumstances exerted an absolute constraint, whereas for those who did, they served to limit the range of options available:

*I’ve tried…but there is only a couple of routes we can take to school so you cannot do too much. I looked at getting the bus and walking to work and back but I’ve got such a small time period to get back from work, pick the kids up, I just cannot do it. There aren’t enough hours in the day for me to do it all.* (Participant 10)

*Some days because I have two – one is in nursery not far from* [city ward] *– I cannot physically get one and then the other one and walk, so I have to drive. And what I’ve done is drive halfway to school and then we’ll walk through the woods and we’ll walk back through the woods, get in the car and then drive to the other one.* (Participant 11)

*Cycling, I really enjoy it but I’m also a bit scared of it as well because it’s not very well set up for it. We have seats for the little ones that we use on holiday…but I would never take them on a road in one. I would admit…if the cycle lanes were segregated it would be so much better. We would cycle loads more. I wish I could cycle more than I do, but I do not feel like it’s a safe option necessarily. It’s too dangerous, it’s really dangerous.* (Participant 13)

*There’s no way…whichever way we go there’s a busy road. And there’s no route that can go down the side streets or anything. To get to a side road you have to go down an even busier road. It’s tricky.* (Participant 15)

As can be seen, at this point, more structural constraints of the social environment are coming to bear on participants. Resulting in the emotion-focused response of resignation among both major groups, those who attempted behavioural changes and those who did not, this brings into sharp focus the limits of individual agency within a socio-structural context unconducive to behavioural change aimed at minimising exposure to air pollution.

### General beliefs: uncomfortable awareness and understanding

Although resignation is a negative response, it is important to note that participants also described use of the monitors in positive—albeit qualified—terms. This was because of the effect exerted on their general and specific beliefs about air pollution. Participants widely noted becoming more sensitive to sources of air pollution in the immediate vicinity of the school run, and are aware that air pollution in a given locale can originate from much further afield. Many also reported being more sensitive to news stories on the topic and of air pollution being an issue elsewhere, such as in cities or on journeys unrelated to the school run. Taken together, this amounted to an increasing awareness of air pollution both narrowly and more generally, a response deemed positive for two main reasons. The first was intrinsic, with participants appreciating the data for no other reason than to know. Here, positivity was derived from the way in which the monitors revealed an issue previously hidden from view. The second was extrinsic, with many seeing the data as being able to imbue their claims with credibility should they approach others about pursuing remedial measures, such as those idling in cars, school managers and local politicians. However, while deemed positive, this awareness was also perceived as simultaneously uncomfortable, especially when coupled with the realisation that structural and environmental factors largely beyond individual control were inhibiting their ability to respond effectively to the “threat” of air pollution:

*My experience was positive and negative; a bit of both. So, positive in my own head, but also negative in my own head as well because it makes you aware of something that is not very nice and something that you do not have much control over. Positive because I feel like there are some things I can do about it. Feeling positive if I cycle and negative if I go in the car, feeling guilty. Feeling more cross with other people but then recognising that I do not know why people are in their cars; there could be multiple different reasons. Being cross with the council or the government, but knowing that actually the council cannot do much about it because they have not got any money. So positives and negatives on all levels, really.* (Participant 17)

*It was positive because it’s always positive to learn something. And it was negative in that it was worse than I was expecting. But it was positive that you are more aware and that you are thinking about it more. Ignorance is bliss, is not it? You can pretend it’s not happening when you do not know about it.* (Participant 20)

*It’s positive in the sense that it’s given me a better awareness of air quality and made me think i should think in different ways. Negative, just in the sense of like I say, it makes you aware that there is bad air around school and it’s just a bit sad. It would have been really positive if it had been really green there and we could have thought ‘oh, this is good’, but it wasn’t.* (Participant 34)

Much like that described by Oltra et al. (2017), there is an important distinction to be made between awareness and understanding. While awareness denotes an increasing sensitivity to air pollution issues and its association with certain sources, understanding refers to specific knowledge of air pollutants, their origins and impact on human health. The monitors proved to be highly effective at increasing the former, but less effective at improving the latter. As participant 18 noted, “it has made me more aware, but I also now know how little I know”; a distinction echoed by participant 37 who, using slightly different language, explained that “I’m more aware but not educated”. This difference was present throughout the sample. Compared with responses prior to using the monitor, over half (*n* = 24) reported “no change” in understanding, 44% (*n* = 20) a minor increase and 1 person a decrease as they became aware of the complexity of the issue. Taken together, at the conclusion of the study, 80% (*n* = 36) of participants categorised their understanding of air pollution as either “none” or “slight”. As such, the monitors were effective at raising awareness and sensitising participants to air pollution as an issue, but not so effective at educating users on air pollution in relation to its make-up, origins and effects on human health.

### Specific beliefs: the disruption of perceived sanctuary spaces

While the monitors exerted a more general influence on participant awareness of air pollution as an issue, they also affected more specific beliefs. The two mainly spoken of by participants pertained to the notion of sanctuary: one relating to indoor space and the other to outdoor. Taking the first as a point of departure, almost half of the sample (*n* = 19) experienced a form of surprise when the monitors reported poor air quality in the home. This belief lay dormant and unacknowledged until revealed and then disrupted by the data. Echoing the “home-as-haven” concept, where “private” and “public” tend to be positioned along the respective lines of security and insecurity (Graham et al. 2015), air pollution is here believed to be a phenomenon existing outside of residential space:

*I just keep looking in the house and I was going ‘oh my god, this is in our own house! What am I doing to everybody?’. That alarmed me.* (Participant 5)

*It’s definitely changed the way I view my house as a safe environment. I think as a parent you think ‘okay, we’re home, we’re safe, it’s all fine’. Well I do not think I can believe that anymore.* (Participant 9)

*There was this moment where it was really high in the sitting room, and I’ve got the windows open and I’m checking it and it’s high and I’m thinking ‘what’s going on here?’* (Participant 13)

*I would consider getting an air purifier in my house, because I did not realise how bad it was. That shocked me.* (Participant 14)

Many encountered high levels when cooking meals either side of the school run, or around the time family members were getting ready in the morning. This is because indoor NO_2_ and particulate matter tend to originate from domestic appliances which burn carbon containing fuels, such as boilers, heaters, fires, stoves and ovens. Similarly, VOCs emanate from cleaning and personal care products, building materials and household consumer goods, such as carpets, laminate furniture, air fresheners and cleaning products (Public Health England 2018). However, this was largely unknown to participants, a feature reported elsewhere, with one survey of 2000 adults noting that 46% could not detail any causes of indoor air pollution and only 36% were aware of its effects on health (Whiffen 2018; see also Niphadkar et al. 2009). The reasons for this are not clear, but it may be because research on indoor air pollution is overshadowed by its outdoor counterpart. Indeed, the Royal College of Paediatrics and Child Health (2018: 1; 2019) labels indoor air pollution a “Cinderella subject”, by virtue of its marginalisation, and has initiated a wide-ranging study on its intersection with child health. This may also be related to media coverage which eschews information on the human health effects of indoor air pollution (see Mayer 2012). Whatever the reason, the “home-as-haven” idea rests on this absence of knowledge, which is why the monitoring data served to destabilise it, precipitating a surprise response and a subsequent change in belief.

This bears similarity to the second belief demonstrated, where almost half of the participants (*n* = 22) reported surprise at the levels of air pollution encountered in green spaces. Exposing an important caveat to the “home-as-haven” concept, where not all outdoor spaces are perceived as equally threatening (see Mallett 2004), this “green-is-clean” assumption reveals a belief that trees and plants can remove air pollution at a rate able to mitigate levels harmful to human health. Again, it is only in the presence of the monitoring data that this belief becomes apparent and, in much the same way as the “home-as-haven” idea, undermined:

*I was really surprised when we went down to the park. I was expecting that to be fine…it’s all surrounded by trees and it was still pretty high. I was thinking ‘oh gosh, we can’t escape this!’* (Participant 2)

*We always walk through the woods. It’s safer for the children and it’s more interesting, but also there’s that idea that there’s a bit of a green barrier. But we would be in the middle of the woods and it would still be quite high. I think I was expecting to find areas of sanctuary from it, but I realised that actually on some days there is not, you know? It’s everywhere. So that was surprising to me*. (Participant 13)

*Because our school is set back from the road, it’s amongst a lot of greenery and you think you know the school run is bad but you think that once you are amongst that greenery that somehow that helps but, I mean, honestly…We’ve been campaigning for a green wall at [school name]. They’ve put in what we could afford. Is it good though? Because now we are thinking ‘oh my goodness, is that going to make any difference whatsoever?’.* (Participant 21)

*It was quite concerning because I did not think it would be so bad. Around here it’s quite leafy and green, is not it? Even with the main road I thought ‘well, there’s trees all along it we’ll probably be alright’. But it was worse than I expected.* (Participant 38)

Considering that people who live in areas with more and/or larger street trees report better health perception (Kardan et al. 2015), and Sheffield boasts more trees per person than any other city in Europe (Styles 2011), the presence of this belief is somewhat understandable. Buttressed by national news stories noting the mitigating effect of tree planting on climate change (see Carrington 2019; England 2019), these beliefs are reflected in a growing body of research demonstrating a relationship between positive experiences and time spent in greener environments. For instance, it is associated with the prevention and mitigation of stress, anxiety and depression for both adults and children (Sefcik et al. 2019; Beyer et al. 2014; Maas et al. 2009; Berman et al. 2008), particularly in urban areas (Razani et al. 2018; Fair et al. 2017; Thompson et al. 2012). There are also case-specific features to account for. Sheffield has, for almost half a decade now, been a site of well-publicised tension over the city council’s felling of street trees. Without recounting the situation in detail (see Heydon 2020), the media coverage following the initial “dawn raid” by police on elderly protestors, and subsequent high-profile advocacy campaigns by community groups, does mean that Sheffield citizens have been exposed to a disproportionate amount of information relating to the benefits of street trees. Many of the participants mentioned this ongoing conflict during the interviews.

Whatever the specific origin of this “green-is-clean” belief, the monitors both reveal its presence and subsequently undermine it, again bringing the complexities of air pollution to bear on participant assumptions. Trees can remove gaseous air pollution through uptake (Setälä et al. 2013; Nowack et al. 2006), and act as a barrier by retaining particles on the plant surface (Tong et al. 2016), but they can also have an adverse effect on air quality. Not only can certain species emit volatile organic compounds (Vivaldo et al. 2017), but vegetation is of little benefit for reducing nitrogen dioxide in urban areas, can exacerbate the build-up of pollution in street canyons by reducing air flow and is better at redistributing air pollution than removing it (National Institute for Health and Care Excellence 2019; Air Quality Expert Group 2018). The monitors served to alert participants to the “green-is-clean” belief before disrupting it, making them attentive to this more complicated reality and contributing to their increased awareness of the air pollution issue at hand.

## Discussion

There are several ways in which personal air quality monitors influence individual thought and action around environmental risk. The technology works to alter the behaviour of users by making a previously imperceptible risk visible. On becoming visible, preconceptions about the “threat” it poses to what is “meaningful”, which in this case is the health of children, are confirmed. This intersects with the idea that individual action can manage such a “threat”, providing the initial impetus to consider undertaking changes aimed at minimising the exposure of children to air pollution on the school run. To a large extent, this confirms the findings of existing studies, where individual attempts at avoiding or mitigating air pollution have also been witnessed in users of similar technology (Bales et al. 2019; Wong-Parodi et al. 2018; Zappi et al. 2012). Where the findings diverge is in relation to the factors implicated in, and the influences acting upon, the decision-making pathways underpinning this decision-making process, a divergence that adds to existing understandings in three key respects.

First, the findings show that sensor technology does not generate a simple binary response among users—behavioural change (Bales et al. 2019; Wong-Parodi et al. 2018) or not (Oltra et al. 2017)—but instead shows it to be capable of producing both in the same user over the same period. Over time, as attempted behavioural changes fail to produce the improvements in exposure expected, and the range of options available for pursuing effective change starts to narrow, negative feelings can colour users’ increased awareness of air pollution with discomfort and risk eliciting an eventual inclination towards inaction. That said, it is not difficult to see how this relationship could also move the other way, with users settling on emotion-focused coping at the outset but pursuing more problem-focused efforts as their circumstances change (e.g. if air pollution in a region is less pervasive, they move to another house, or their children progress to more senior schools that are further away).

Second, monitor use is capable of altering beliefs about air pollution independent of behavioural change. This was seen in relation to the ability of monitors to reveal and subsequently disrupt misconceptions about indoor and outdoor “sanctuary spaces”. Further, alerting users to the pervasiveness of air pollution both generally and specifically, this exerted influence at the point of reappraisals by introducing a perception of inescapability into user beliefs; a reaction intrinsically linked to the reported feelings of powerlessness. This holds relevance because existing literature has given disproportionate priority to the influence of monitors on behavioural change. Yet, their ability to alter beliefs is at least as important because such cognitive configurations are heavily implicated in behaviour change. As Lazarus and Folkman (1984: p. 63) take care to note, beliefs are “pre-existing notions about reality which serve as a perceptual lens…determining what is fact, that is, “how things are” in the environment, and they shape the understanding of its meaning”. With perceptions acting as a filter through which external reality is experienced, further exploring the effect of personal monitors on this aspect of cognition is a key to understanding their influence on human behaviour.

Third, existing studies conceptualise monitor use in terms of a simple dualistic relationship between technology and individual. However, as can be seen here, it is more accurate to conceive of the relationship as tripartite: between monitor, individual and socio-structural context. Indeed, the extent to which behaviour change continues over time is largely determined not by the agency of individuals, but by the various socio-structural circumstances with which they are entwined. As noted above, the main difference between the two broad groups of participants—those who pursued change from the outset and those who did not—was the point at which these circumstances converged to produce strategy asphyxiation during secondary appraisal. The associated feelings of powerlessness and resignation cannot therefore be said to originate in the monitoring technology itself, but in the social structure that limits what users can do with the information provided by it. Existing literature has highlighted the limits personal efforts have on either the degree of exposure or level of emissions present in a given area, largely because of their complications and partial efficacy when compared with measures targeting emissions at source (Laumbach et al. 2015), but these are different to the temporal and spatial constraints reported by participants here. As such, this study draws attention to the importance of external social factors when determining internal human responses to monitoring data. Indeed, it is for this reason that, when considering the influence of personal monitoring technology on decision-making, socio-structural context cannot be seen as peripheral to the process, but integral to it.

## Conclusion

Taken together, the study has demonstrated that personal environment monitors can play a role in protecting children from air pollution on the school run. They are effective at raising awareness about air pollution, disrupting misconceptions about where it does and does not occur, and encouraging users to change their behaviour in an attempt to mitigate and manage the risks. However, their ability to produce lasting and effective behaviour change is stymied by socio-structural constraints. As such, it is only with top-down support aimed at tackling air pollution at source that this bottom-up technology will attain its full potential.

This has several implications for advancing personal environment monitors and for future research on their social consequences, three of which will be emphasised here. With regard to the former, the social dimension of the technology itself requires further development. Currently, the monitors mistakenly represent public issues as private problems. The more this technology can encourage cooperation between interested individuals then the likelihood for effective collective action—that is, a social response to social-structural constraints—is magnified. Similarly, the data collected could be integrated with projects aimed at collating this data and making it publicly available, an approach already underway at the Urban Flows Observatory at the University of Sheffield (2018). The technology also needs to improve its educational dimension, in terms of the accessibility, in order to capitalise on its effective awareness-raising role. With regard to the latter, further research is needed into the long-term effects of this technology not only on beliefs and behaviours, but also on how decision-making patterns differ between populations embedded in a range of socio-structural circumstances. Only then can a full appreciation of the transformative potential of this technology for a variety of user groups be fully understood.
